# Receptor Guanylyl Cyclases in Sensory Processing

**DOI:** 10.3389/fendo.2016.00173

**Published:** 2017-01-11

**Authors:** Ichiro N. Maruyama

**Affiliations:** ^1^Information Processing Biology Unit, Okinawa Institute of Science and Technology Graduate University, Okinawa, Japan

**Keywords:** behavior, *C. elegans*, dauer formation, gustation, olfaction, phototransduction, thermosensation

## Abstract

Invertebrate models have generated many new insights into transmembrane signaling by cell-surface receptors. This review focuses on receptor guanylyl cyclases (rGCs) and describes recent advances in understanding their roles in sensory processing in the nematode, *Caenorhabditis elegans*. A complete analysis of the *C. elegans* genome elucidated 27 rGCs, an unusually large number compared with mammalian genomes, which encode 7 rGCs. Most *C. elegans* rGCs are expressed in sensory neurons and play roles in sensory processing, including gustation, thermosensation, olfaction, and phototransduction, among others. Recent studies have found that by producing a second messenger, guanosine 3′,5′-cyclic monophosphate, some rGCs act as direct sensor molecules for ions and temperatures, while others relay signals from G protein-coupled receptors. Interestingly, genetic and biochemical analyses of rGCs provide the first example of an obligate heterodimeric rGC. Based on recent structural studies of rGCs in mammals and other organisms, molecular mechanisms underlying activation of rGCs are also discussed in this review.

## Introduction

**Figure 1:**
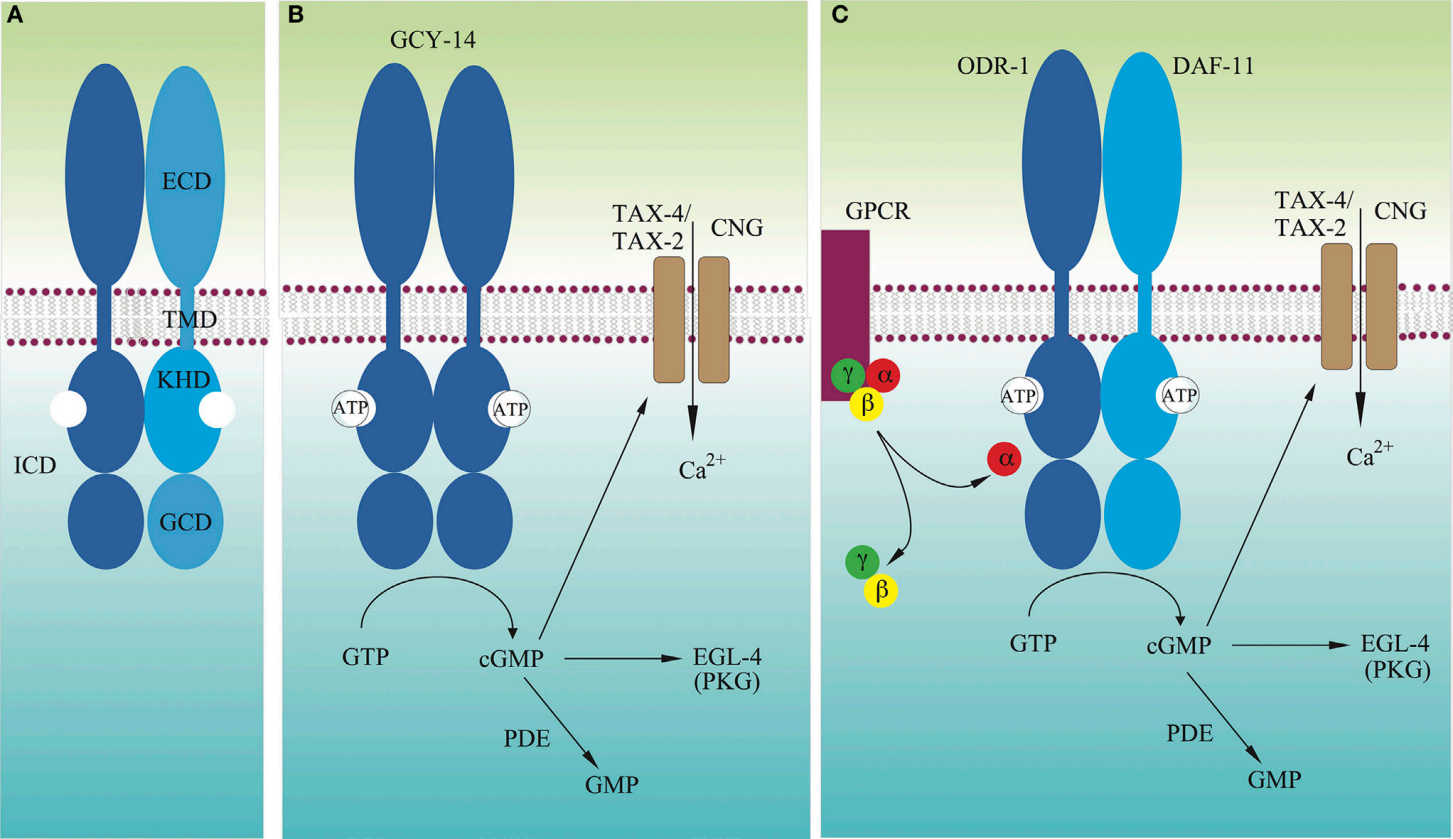
**Models for molecular mechanisms of transmembrane signaling by GCY receptors**. **(A)** Domain structure of typical GCY receptors. **(B)** Many GCYs function as homodimers, and their extracellular domains directly detect extracellular signals, which activate intracellular guanylyl cyclases. **(C)** There are GCYs that act as heterodimers such as DAF-11/ODR-1. Some extracellular cues are recognized by G protein-coupled receptors, which either stimulate or inhibit activity of GCYs. Not drawn to scale.

An intracellular second messenger, guanosine 3′,5′-cyclic monophosphate (cGMP), was first identified in rat urine (1). Since then, it has been demonstrated that cGMP participates in a wide range of physiological responses, including blood pressure regulation, phototransduction, olfaction, thermosensation, and synaptic plasticity (2, 3). cGMP is also important in the invertebrate nervous system. It is involved in ecdysis and foraging behaviors in insects, and in neuronal path finding and differentiation in insects and mollusks (4). In 1969, it was found that both the water-soluble and particulate fractions of tissue homogenates showed guanylyl cyclase (GC) (also called as guanyl cyclase or guanylate cyclase) activity (5–7). The enzyme activity differed in the two fractions (8–10) and was subsequently purified from both fractions (11, 12). The cDNAs of both were cloned (13–16), and GCs are classified into two groups: receptor guanylyl cyclase (rGC, also called membrane GC or receptor-type GC) and soluble GC (sGC) (2, 17). The soluble forms have been shown to exist as heterodimers, consisting of α and β subunits, and containing heme as a prosthetic group. Heterodimerization is required for their catalytic activity (18, 19), although β subunits have been reported to form active homodimers (20, 21). While sGC has a cytoplasmic heme-binding domain and a guanylyl cyclase domain (GCD), rGCs are type-1 transmembrane receptors, comprising of an extracellular domain (ECD), a transmembrane domain (TMD), and an intracellular domain (ICD), which consists of the protein kinase-homology domain [KHD; also called a protein kinase-like domain, or adenosine 5′-triphosphate (ATP)-regulatory module] and a GCD, separated by a ~50 residue linker region (Figure 1A). Apart from the overall structural differences, GCD monomers of sGCs and rGCs have the same protein fold as the mammalian adenylyl cyclase (AC) catalytic domain (3). rGCs can be activated either by extracellular ligands, such as natriuretic peptides and uroguanylin in GC-A, GC-B, and GC-C, or by intracellular calcium-binding proteins, such as guanylyl cyclase-activating proteins (GCAPs) in GC-E (also known as RetGC-1) and GC-F (RetGC-2) (2, 17, 22).

The *Caenorhabditis elegans* genome encodes 27 rGCs (Table 1) (23–25). These numbers are unusually large, compared to other genomes. For example, mammalian and *Drosophila* genomes encode 7 and 6, respectively (4). Recent studies have elucidated physiological roles of rGCs in *C. elegans*, on which we focus in this review. Ectopic expression of rGCs in different neurons has shown that rGCs play roles as direct sensor molecules for alkaline pH, molecular CO_2_, and temperature in sensory neurons (26–28). Many other rGCs are also involved in salt gustation, olfaction, pheromone detection, phototransduction, and body-size regulation. Based on recent structural studies of rGCs in mammals and other organisms, molecular mechanisms underlying activation of rGCs are also discussed. rGCs of other organisms have been reviewed elsewhere (2–4, 17, 22, 29).

**Table 1:** **Expression and function *of Caenorhabditis elegans* receptor guanylyl cyclases**.

Receptor guanylyl cyclase (rGC)	Function	Reference	Cell/neuron expressing rGC	Reference
ODR-1 (GCY-10)	Olfaction, dauer formation, phototransduction	(30–32)	ASIL/R, ASJL/R, ASKL/R, AWBL/R, AWCL/R	(24, 31)
DAF-11	Olfaction, dauer formation, phototransduction	(30, 31, 33)	ASIL/R, ASJL/R, ASKL/R, AWBL/R, AWCL/R	(33)
GCY-1	K^+^	(34)	ASER, ASIL/R, PVT, URXL/R, AIYL/R, intestine	(23)
GCY-2			ASIL/R, AWAL/R, RIAL/R, PVT	(23)
GCY-3			ASER, ASIL/R, PVT	(23)
GCY-4	Br^−^, I^−^	(34)	ASER (biased)	(24)
GCY-5			ASER	(24)
GCY-6	Mg^+^	(34)	ASEL	(24, 35)
GCY-7			ASEL, excretory canal cell (only in adults)	(23, 24, 36)
GCY-8	Thermosensation	(28, 37)	AFDL/R	(24)
GCY-9	Carbon dioxide	(27)	BAG	(23)
GCY-11			Pharyngeal muscle	(23)
GCY-12	Body size	(38)	ASE, AWC, ASJ, AUA, PHAL/R, PHB	(24, 38)
GCY-13			RIML/R	(23)
GCY-14	Na^+^, Li^+^, Alkaline pH	(26, 34)	ASEL (biased), AWCL/R (faint), PVT	(23)
GCY-15			ASGL/R (faint)	(23)
GCY-17 (GCY-24)			PHAL/R	(23)
GCY-18 (GCY-26)	Thermosensation	(28, 39)	AFDL/R, AIML/R	(23)
GCY-19			ASEL/R (faint), IL2, additional three sensory neurons (faint)	(23)
GCY-20 (GCY-16)			ASEL, AWCL/R (faint), excretory gland and canal cells	(23)
GCY-21			ASGL/R, ADLL/R (faint)	(23)
GCY-22	Nearly all salts, methionine	(34, 35, 40)	ASER	(36)
GCY-23	Thermosensation	(28, 37)	AFDL/R	(23, 37)
GCY-25			AQR, PQR, URXL/R	(23)
GCY-27			ASKL/R, ASIL/R, ASJL/R	(23)
GCY-28	Olfaction, behavioral choice	(41, 42)	Many neurons, muscle, hypodermis, somatic gonad, intestine	(23, 42)
GCY-29			AFDL/R, ASEL/R, AWCL/R, AVKL/R, few other neurons (faint)	(23)

## Roles of rGCs in Sensory Processing

### Gustation

**Figure 2:**
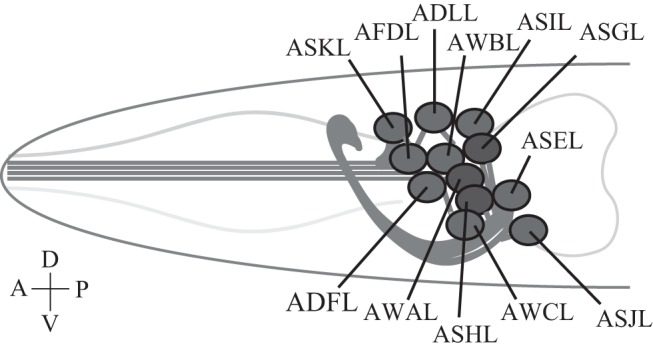
***Caenorhabditis elegans* sensory neurons**. A subset of *C. elegans* amphid sensory neurons described in this review consists of a pair of two bilaterally symmetric neurons. Each of the 12 pairs of neurons extends a dendrite to the tip of the nose, and an axon into the nerve ring, a nerve bundle where synaptic connections are made with other neurons including downstream interneurons (51). Only left-side neurons are shown. Not drawn to scale.

A bilaterally symmetric pair of *C. elegans* gustatory neurons, left ASE (ASEL) and right ASE (ASER) (refer to Figure 2 for the positions of sensory neurons described in this review), senses a number of chemicals in a left/right asymmetric manner and coexpresses multiple rGCs, GCY-6 (Guanylyl CYclase), -7, -14, -19, -20, and -29, and GCY-1, -3, -4, -5, -19, -22, and -29, respectively (23). GCY-14 of ASEL is essential in sensing environmental alkaline pH (26). Ectopic expression of GCY-14 in other sensory neurons, ASG, ASI, and ASER, makes these neurons sensitive to alkaline pH. GCY-14 functions as a homodimer, like mammalian rGCs (Figure 1B). Histidine-174 of the GCY-14 ECD is required for the detection of alkaline pH. Deprotonation of this histidine residue by alkaline pH may cause conformational changes in the domain that activates intracellular GC. Activation of GCY-14 then opens cGMP-gated cation channels consisting of TAX-4 (abnormal chemoTAXis) (α) and TAX-2 (β) subunits (43, 44), resulting in Ca^2+^ entry into ASEL. This Ca^2+^ entry also involves EGL-4 (EGg Laying defective), a cGMP-dependent protein kinase (PKG) (45–47), TAX-6, a calcineurin A ortholog (48), and phosphodiesterases (PDEs) (30). A neuronal calcium sensor (NCS-1) (49), which is a calcium-binding protein related to vertebrate GCAPs and 74% identical to human frequenin (50), is not required for Ca^2+^ entry, but enables its downstream signaling, since chemotaxis of *ncs-1* mutants to alkaline pH is deficient (26).

GCY-14 is also required for sensing increases in Na^+^ or Li^+^ concentrations (34) and is a direct sensor for an increase in NaCl concentrations (26). In contrast to alkaline pH sensation, histidine-174 does not play a role in detecting increases in NaCl concentration, suggesting that other residues of the ECD are responsible for sensation. Disruption of the ASER-expressed rGC gene, *gcy-22*, results in broadly defective chemotaxis to nearly all salts sensed by ASER (27, 34, 40). *gcy-22* is also required for animal responses to the amino acid, methionine, which is primarily sensed by ASER (34). In contrast, disruption of other *gcy* genes results in highly salt-specific chemosensory defects (34). *gcy-1* mutant animals show markedly decreased responses to K^+^, which is sensed by ASER. *gcy-4* shows chemotaxis defects on gradients of Br^−^ and I^−^. *GCY-4* and *GCY-22* may exist as homodimers and heterodimers, with each homodimer retaining residual function, since a *gcy-4;gcy-22* double mutant shows a stronger defective phenotype in chemotactic response to Br^−^ and I^−^ (34). Although all mammalian rGCs exist in a homodimeric form, but not in a heterodimeric form, it has been shown theoretically and experimentally that *C. elegans* rGCs, ODR-1 (ODoRant response abnormal) and DAF-11 (abnormal DAuer Formation), function as an obligate heterodimer (4, 26). *gcy-6* mutant animals show a dramatic decrease in their ability to respond to Mg^+^ gradients, sensed by ASEL (34). However, except for GCY-14, it remains to be shown whether these GCYs are direct sensor molecules by ectopic expression of these rGCs in unrelated neurons.

### Thermosensation

*Caenorhabditis elegans* exhibits defined behavioral responses to thermal gradients, which evoke two distinct behaviors. The first is cryophilic movement, or migration toward cooler temperatures than the growth temperature. This is the dominant behavior when the ambient temperature exceeds the growth temperature and is achieved by means of a biased random walk (52–54). The second behavior, isothermal tracking, is observed when animals reach thermal zones that are within 2°C of the growth temperature (52, 54–57). These behaviors involve at least three pairs of sensory neurons, AFD, AWC, and ASI (53, 56, 58, 59). AFD neurons, whose sensory dendrites terminate in a specialized ending that is composed of a primary cilium and an extensive array of microvilli (51, 60), depolarize and hyperpolarize upon warming and cooling, respectively, and temperatures warmer than the growth temperature drive cryophilic thermotactic behaviors (61). The thermosensory responses appear to be AFD cell-intrinsic properties, although surrounding glial cells tune thermal responses of AFD (62, 63).

At least eight genes are essential for thermotransduction by AFD: *gcy-8, gcy-18, gcy-23, tax-4, tax-2, pde-2*, and *ncs-1*. The three rGCs, GCY-8, GCY-18, and GCY-23, are exclusively expressed in AFD (37, 64), and participate in isothermal tracking (59, 65) and migration in linear thermal gradients (66). Recently, it has been shown by ectopic expression in diverse cell types that these rGCs are indeed direct thermosensor molecules, and that both the extracellular and ICDs are necessary for sensation (28). The TAX-4/TAX-2 cGMP-gated cation channel is also expressed in AFD and is essential for thermotaxis (37, 43, 44, 61). In AFD, *pde-2* and *pde-5*, but not *pde-1* or *pde-3*, are expressed, and *pde-2*, but not *pde-5*, mutations increase the threshold temperature for the activation of thermoreceptors and augment the thermoreceptor current lifetime (67). NCS-1 is also expressed in AFD and regulates behavioral responses to thermal gradients (49). As in *pde-2* mutants, loss of NCS-1 prolongs thermoreceptor currents and elevates their threshold. Unlike loss of PDE-2, however, loss of NCS-1 increases voltage-activated outward currents (67). These results suggest that cGMP concentrations and NCS-1 help set the threshold temperature of rGCs in AFD.

*Caenorhabditis elegans* AMsh glial cells ensheathe neuronal receptive endings of 12 neurons, including AFD. It has been recently shown how glia control shapes of neuronal receptive endings through inhibition of an rGC expressed on the neuronal surface (68). KCC-3, a K^+^/Cl^−^ cotransporter, localizes specifically to the glial microdomain surrounding AFD receptive ending microvilli, where it regulates K^+^ and Cl^−^ levels. Cl^−^ ions act as a direct inhibitor of the GCY-8 rGC on the AFD receptive ending microvilli *via* Cl^−^ binding to the receptor’s ECD. GCY-8 has basal activity, which is inhibited by extracellular Cl^−^ ions released from AMsh glial cells. Without the inhibition, an increased level of cGMP at the neuronal receptive ending promotes the disappearance of AFD receptive ending shapes. Similarly, a *pde-1 pde-5* double mutant displays a fully penetrant loss of AFD receptive endings. PDE-1 and PDE-5 are the only PDEs required for extension of AFD microvilli and appear to function redundantly. It appears that higher levels of cGMP at the AFD neuronal receptive ending antagonize WSP-1/Wiskott–Aldrich syndrome protein, an actin regulator that promotes microvillus formation through nucleation of actin filaments in the receptive ending (68). However, it remains to be clarified how cGMP antagonizes WSP-1 activity.

### Carbon Dioxide Detection

Carbon dioxide is detected by animals as an environmental cue that indicates the presence of food, predators, or mates, and as an internal cue that reflects internal metabolic state (69, 70). *C. elegans* has a pair of CO_2_-sensing neurons, the BAG neurons, which mediate avoidance of CO_2_ by adults and attraction to CO_2_ by dauer larvae (71, 72). Adult *C. elegans* animals display an acute avoidance response upon exposure to CO_2_ that is characterized by cessation of forward movement and rapid initiation of backward movement. This CO_2_ avoidance is mediated by a cGMP signaling pathway that includes the cGMP-gated heteromeric channel TAX-4/TAX-2 (71). The GCY-9 rGC is responsible for CO_2_ sensitivity of the BAG neurons (27). CO_2_ avoidance behavior is modulated by multiple signaling molecules, including the neuropeptide Y receptor, NPR-1, and the calcineurin subunits, TAX-6 and CNB-1 (CalciNeurin B) (71). GCY-9 is a direct sensor for molecular CO_2_, since the receptor ectopically expressed in AFD thermosensory neurons, which normally do not respond to CO_2_, responded to CO_2_ (27).

### Olfaction

*Caenorhabditis elegans* uses two pairs of ciliated olfactory neurons, AWA and AWC, to sense many volatile attractants (39). A pair of laterally symmetric AWC neurons, AWCL and AWCR, are functionally distinct from each other. The distinction between the two AWC neurons is random from animal to animal, but coordinated so that one neuron of each type is generated in each animal (73). AWC^on^, which expresses the STR-2 (Seven Transmembrane Receptor) G protein-coupled receptor (GPCR), detects butanone, benzaldehyde, and isoamyl alcohol, whereas AWC^off^, which does not express STR-2, detects 2,3-pentanedion, benzaldehyde, and isoamyl alcohol (74). Chemosensation in AWC is mediated by direct binding of odorants to GPCRs, which are exclusively localized to sensory cilia (75, 76). Intracellularly, these receptors are coupled to heterotrimeric G proteins, whose α subunit can positively or negatively regulate *C. elegans* chemotaxis to odorants (77). There are at least four Gα subunits expressed in AWC: ODR-3, GPA-2 (G Protein, Alpha subunit), GPA-3, and GPA-13 (77–79). Butanone signaling is mediated by two partly redundant Gα proteins, ODR-3 and GPA-2, whereas other odor responses in AWC are mediated by ODR-3, but not by GPA-2 (78). AWC utilizes cGMP as a second messenger, as well as the cGMP-gated cation channel TAX-4/TAX-2. ODR-1 and DAF-11 rGCs are both required for the animal’s chemotaxis to all AWC-sensed odorants (31, 33, 39), and these rGCs form obligate heterodimers (Figure 1C), each monomer of which provides essential catalytic residues to form a single catalytic site (4, 26). The ECD of ODR-1 is dispensable for its olfactory function, indicating that the rGC does not act as a direct receptor for odorants sensed by AWC (31).

Genetically encoded calcium indicators have revealed that in the absence of odorants, the AWC exhibits high intracellular calcium levels, and upon odorant stimulation, intracellular calcium levels decrease, leading to hyperpolarization of the AWC neurons (80). This hyperpolarization can be explained by the decrease of cGMP levels followed by closing of TAX-4/TAX-2 channels. This mechanism shares many similarities with the light response in mammalian photoreceptor cells, where in the absence of light, calcium channels are open, exhibiting a “dark current.” Upon photon binding, a cGMP PDE is activated, which reduces cGMP levels, closing cGMP-gated channels, and thus hyperpolarizing the cell (81). Therefore, in AWC olfactory sensory processing in *C. elegans*, GPCRs act as sensor molecules, and upon stimulation, release their Gα subunits to regulate cGMP concentration in AWC cilia. Interestingly, *C. elegans* chemotaxis to odorants sensed by AWC is not affected by loss of the PDEs (82). Therefore, the released Gα may directly interact with the heterodimeric ODR-1/DAF-11 rGC to lower the GC activity (Figure 1C) (31).

The EGL-4 PKG is necessary for adaptation of the AWC chemosensory response (47, 83). Nuclear localization of EGL-4 is both necessary and sufficient to promote long-term adaptation (84) and is dependent on PDE activity (82). ODR-3, a Gα subunit, and the ability of EGL-4 to bind cGMP are both required for nuclear entry of EGL-4 after prolonged odorant exposure. Furthermore, loss of ODR-1 leads to constitutive entry of EGL-4 into the nucleus (83). Nuclear EGL-4 phosphorylates HPL-2 (Heterochromatin Protein Like), a heterochromatin-binding protein, and promotes the phosphorylated protein to bind to the *odr-1* locus in AWC in order to reduce *odr-1* mRNA levels in adapted animals. Concomitantly, the increased activity of an endo-siRNA pathway-targeted *odr-1* degrades the mRNA (85).

An attractive behavior mediated by AWC^on^ requires the GCY-28 rGC, which acts in adults and localizes to the AWC^on^ axon. Mutations in *gcy-28* lead to an avoidance behavior instead of the attractive behavior normally directed by the AWC^on^ neuron. This behavioral reversal results from presynaptic changes in AWC^on^ possibly through modification of AWC^on^ excitability or synaptic release by GCY-28 (41). The *gcy-28* mutants also show an abnormal bias in the behavioral choice between two conflicting cues, the attractive odorant diacetyl sensed by AWA and the aversive stimulus Cu^2+^ sensed by ASH/ADL, although their responses to each individual cue are similar to those in wild-type animals (42). GCY-28 regulates the neuronal activity of AIA interneurons, where the conflicting sensory cues from AWA and ASH/ADL sensory neurons seem to converge, by activation of CNG-1, a cyclic nucleotide-gated ion channel (42, 86).

### Dauer Formation

The environment determines whether *C. elegans* grows directly into adulthood, or whether it arrests at an alternative L3 larval stage to form a dauer larva (87). Dauer larvae are induced by harsh conditions, such as starvation and high population density, and can survive under severe conditions because of distinctive morphology, metabolism, and life span. When environmental conditions improve, dauer larvae reenter the reproductive cycle by molting into L4 larvae and subsequently into adulthood.

The dauer larva versus reproduction and growth decision is determined by at least two signaling cascades: the DAF-2 (insulin/IGF-1 receptor) (88) and the DAF-7 (TGFβ) pathways (89, 90). A decrease in either of the signals causes dauer arrest, indicating that both pathways are required for reproduction and growth. Downregulation of DAF-2 results in the activation and nuclear localization of DAF-16 (forkhead transcription factor) (91–93). The DAF-2 signaling pathway also regulates metabolism and aging. When DAF-2 signaling is decreased, life span is greatly extended (94, 95). *daf-7* encodes a member of the TGFβ superfamily that is a ligand for the parallel neuroendocrine pathway (89, 90). DAF-7 activates a heterodimeric receptor consisting of DAF-1 (TGFβ type I receptor) (96, 97) and DAF-4 (TGFβ type II receptor) (98).

Laser ablation of three sensory neurons, ADF, ASG, and ASI, causes dauer arrest, indicating that these neurons signal reproduction and growth (99). Mutations in *daf-11*, which is expressed in ciliated sensory neurons including ASI, ASJ, ASK, AWB, and AWC, cause dauer arrest (33). Moreover, cGMP-gated channels have also been implicated (43, 44, 100). Because dauer arrest caused by a loss-of-function mutation, *daf-11(lf)*, can be partially suppressed either by *daf-3(lf)* or *daf-16(lf)*, DAF-11 is likely to regulate both the DAF-2 and DAF-7 pathways (101, 102). Furthermore, dauer-inducing pheromone inhibits DAF-7 expression and promotes dauer arrest, and food activates DAF-7 expression (89, 90). *daf-7* gene expression is defective in *daf-11* mutants, and the constitutive dauer formation phenotype of *daf-11* mutants is suppressed by DAF-7 expression in ASI (103).

Two GPCRs, SRG-36 (Serpentine Receptor, class Gamma) and SRG-37, are strongly expressed in ASI neurons, where they localize to the sensory cilia, as receptors for the dauer pheromone, ascaroside ascr#5 (32, 104). A heterodimeric GPCR, DAF-37/DAF-38, is expressed in ASI and functions as a receptor for ascr#2 (105). *odr-1* mutants have reduced sensitivity to dauer pheromone, indicating a role in ascaroside perception (32). While *egl-4(lf)* mutations increase the propensity to form dauer larvae (106), *egl-4(gf)* mutations decrease that propensity (107).

Taken together, at high population density, concentrations of dauer pheromone ascarosides increase and activate GPCRs on the cilia of ASI and other neurons. Gα released from the receptors may interact with heterodimeric ODR-1/DAF-11 rGC to regulate GC activity.

### Phototransduction

*Caenorhabditis elegans* lives in darkness, but is able to sense blue or shorter wavelength of light (with maximal responsiveness to ultraviolet light), and engages in negative phototactic behavior (108). This negative phototaxis is important for survival because ultraviolet and blue light are toxic to the animal on the soil surface and requires *lite* (light-unresponsive) genes (108). Laser ablation of a combination of four ciliated neurons (ASJ, AWB, ASK, and ASH) led to a severe defect in the negative phototaxis from light (109).

*Caenorhabditis elegans* phototransduction requires LITE-1, a seven-transmembrane domain receptor-like protein that transduces light signals in ASJ *via* G protein signaling (30, 110). *C. elegans* Gα proteins, GOA-1 and GPA-3, have a redundant role in mediating phototransduction in ASJ. Downstream of the Gα proteins, the rGCs ODR-1 and DAF-11 are required for phototransduction in ASJ. cGMP-gated cation channels consisting of TAX-4/TAX-2 act downstream of ODR-1/DAF-11. In the *pde-1, -2*, and -*5* triple mutant, the photocurrent is markedly potentiated, with a current density about fivefold greater than that in wild-type animals. The photocurrent in *pde-1, -2, -3*, and *-5* quadruple mutants exhibits very slow or no recovery after cessation of the light stimulus, consistent with a role for PDEs in downregulating cGMP levels. These results are reminiscent of vertebrate photoreceptor cells, where light-activated G proteins either inhibit PDEs (e.g., parietal eye photoreceptor cells) or stimulate them (e.g., rods and cones), increasing or decreasing cGMP levels and opening or closing cGMP-gated cation channels, respectively (30).

### Regulation of Body Size

Many mutants that lack normal sensory cilia exhibit small body size, suggesting that sensory cilia are involved in regulation of body size in *C. elegans* (111). To elucidate molecular mechanisms underlying sensory regulation of body size, a genetic screen for suppressors of the small body size of a cilium-defective mutant, *che-2* (abnormal CHEmotaxis), which is expressed in the cilia of most ciliated sensory neurons and which encodes a protein that contains G protein β-like WD-40 repeats has been carried out (112). Through the genetic screen, mutants defective in *daf-25, egl-4*, and *gcy-12* have been isolated as suppressors of *che-2* mutations. EGL-4 appears to regulate body size by functioning in sensory neurons, because EGL-4 expression in ASE (and AWC) sensory neurons is sufficient for the animal’s growth to a normal body size (38, 45). *dbl-1* (DPP/BMP-Like) mutants have a small body size with low hypodermal ploidy. The DBL-1 (TGFβ) signaling pathway, which regulates hypodermal ploidy and consequently affects cell growth and body size (113), acts downstream of EGL-4 (45, 46). Indeed, *che-2* and *egl-4* mutants exhibit low and high ploidy of the hypodermal cells, respectively (114).

As described above, mutations in *gcy-12* suppress the small body size of the *che-2* mutant. As observed in *egl-4* mutants, *gcy-12* mutants showed increased body size, compared with wild-type animals (38). However, *gcy-12;egl-4* double mutants did not become larger than *egl-4* single mutants, indicating that *gcy-12* and *egl-4* act in the same pathway. The overexpression of GCY-12 in wild-type animals led to a small body size, presumably due to activation of EGL-4 kinase because GCY-12 overexpression did not cause body-size reduction in *egl-4* mutants (38). These results also suggest that GCY-12 acts upstream of EGL-4, most likely by synthesizing cGMP. However, the ECD of GCY-12 is not necessary for GCY-12 function in body-size regulation (38). The *pde-2* gene, which encodes a PDE is also required for normal body size, and *pde-2* mutations cause a small body-size phenotype. The *pde-2* mutation did not affect the body size of *egl-4* mutants, indicating PDE-2 acts upstream of EGL-4. The *gcy-12;pde-2* double mutants exhibited an intermediate body size relative to the two single mutants. These results indicate that PDE-2 hydrolyzes cGMP and negatively regulates EGL-4 kinase in regulating body size (38). A gain-of-function mutation of *egl-4* that produces a constitutively activated EGL-4 kinase, exhibited small body size, an opposite phenotype of a loss-of-function mutant of *egl-4* (115).

These results suggest the existence of other sensor molecules in addition to GCY-12 that detects extracellular signals. GCY-12 may be constitutively active in ASE. The extracellular signal may lower cGMP levels in ASE sensory neurons perhaps through activation of PDEs and consequently inhibits EGL-4 kinase. Abnormal activation of EGL-4 by an increased concentration of cGMP or by a mutation may upregulate the secretion of DBL-1 (also known as CET-1) or equivalent ligand molecules from ASE sensory neurons. An increase in DBL-1 concentrations may initiate the Sma/Mab signaling pathway in hypodermal cells, which regulates hypodermal ploidy and consequently affects cell growth and body size. In analogy to the role for EGL-4 in olfactory adaptation, in which loss of ODR-1 rGC leads to constitutive entry of EGL-4 into the nucleus of AWC, loss of GCY-12 rGC may also lead to entry of EGL-4 into the nucleus of ASE, where DBL-1 expression may be regulated.

## Mechanism of Activation of rGCs

Like many other type-1 transmembrane receptors such as receptor tyrosine kinases and cytokine receptors (116, 117), rGCs have a homo- or heterodimeric structure in the absence of bound ligand (26, 118–120). The ECD of rGCs often encodes regions that recognize extracellular cues such as peptides or ions. Furthermore, the ECD of many rGCs, including GCY-8 and GC-A (also called NPRA or NPR-1), has the conserved Cl^−^-binding structure motif, S(x)_n_GPxC, near the dimer interface (68, 119, 120). In GCY-8, Cl^−^ binding inhibits its rGC activity (68), while in GC-A, atrial natriuretic peptide (ANP) ligand binding to the receptor is Cl^−^ concentration dependent (121). Therefore, it appears that Cl^−^ ion binding to the ECD of rGCs induces conformational changes in these receptors.

Adenosine 5′-triphosphate is thought to bind to KHD and to augment GC stimulation by ligand binding (122, 123). The interaction between the KHD and ATP shows positive cooperativity, suggesting that binding of one ATP to one KHD of the dimer would facilitate binding of a second ATP to the other KHD (124). The KHD is normally phosphorylated (125, 126), and its dephosphorylation leads to receptor desensitization (126–128). The KHD of GC-A allosterically regulates both peptide binding to the ECD and activation of the GC in a bidirectional manner. ATP binding to the KHD of the GC-A dimer results in reduced ANP binding to the ECDs and enhanced GC activity (129–131). GC-A lacking the KHD is constitutively active (132). The KHD responds to ANP binding by adopting a conformation that allows direct ATP binding, resulting in release from GC activity repression (124). Therefore, it is clear that in the inactive form of rGC, the KHD exists in a stable dimeric form that inhibits its C-terminal GC activity (Figure 1). Stability of the KHD dimer may be affected by phosphorylation states of the domain. Upon ligand binding to the ECD of rGC, the KHD may assume a relatively flexible dimeric configuration, to which one molecule of ATP binds. This first ATP binding may induce configuration changes in the KHD dimer to increase its affinity for the second ATP molecule. Flexible structure of the KHD dimer may allow configuration changes in the GC dimer for activation. Configuration changes in the KHD dimer may also induce configurations of the ECD dimer, in which the ligand has low-affinity binding.

As described above, the KHD is connected to the GCD by a ~50 residue linker region in the receptor’s ICD. Systematic mutational and biochemical analyses of GC-C and GC-A have suggested an important role for the linker region in repressing the catalytic activity of the receptors in the absence of their ligands (133). Specific residues in the linker region seem to assist in repressing GC activity through its interaction with the GCD, where Gα may also interact with and activate (or inhibit) rGC.

Crystal structures of three catalytic domains of a sGC, Cyg12, from the green algae, *Chlamydomonas reinhardtii* (134), of a putative rGC, Cya2, from the cyanobacterium *Synechocystis PCC6803* (135), and of a human sGC α1 and β1 subunits, encoded by *GUCY1A3* and *GUCY1B3* genes, respectively (136), have recently been determined. Furthermore, GCs and ACs belong to the class III nucleotide cyclase family and share high sequence similarity (137). By structural comparison, the dimer structure of Cyg12 GCD is similar to the open, inactive conformation of the AC catalytic domain, to which it must be close in order to be catalytically active (134). On the other hand, the Cya2 GCD dimer is in a closed conformation that must open to bind the substrate GTP (135). Comparison of an “open” apo-AC structure (138) with a “closed” ligand-bound AC structure shows a 7° rotation of the monomer (139). This movement brings the catalytic residues on one subunit closer to the catalytic residues on the other subunit, thereby forming the catalytically competent active site. Comparing the open conformation of the *Chlamydomonas* structure with the closed conformation of the cyanobacteria homodimeric GCD shows a similar, 7°–8°, domain rotation (136, 140). The heterodimeric catalytic domains of the α and β subunits are in an inactive open conformation, but can be superposed onto an active AC by a structural transition involving a 26° rigid-body rotation of the α subunit (136). These structural studies suggest that the GCD subunit monomer of the inactive open structure of the dimer rotates to form an active closed structure. Such a flexible transition between open and closed structures may be essential for GTP binding to and the release of cGMP from the active site of the domain dimer.

As described above, rGCs exist as dimers, like many other type-1 transmembrane receptors (116, 117). ANP binding to GC-A induces a 40° rotation (or twist) of the receptor’s TMD parallel to the plane of the membrane (141). The subunits of GCD dimers also rotate to form a catalytically active structure as described above (136). All these results suggest that upon ligand binding to the ECD of rGCs, the receptor subunits rotate to activate the GCD of the receptor dimer as previously proposed (140). In this transmembrane signaling process, the KHD may play a regulatory role by binding to the allosteric effector ATP and by varying its phosphorylation state. Prior to ligand binding, the KHD may exist as a stable dimer, like the inactive back-to-back homodimeric structure of the kinase domains of the epidermal growth factor receptor (142, 143). Like many other type-1 transmembrane receptors (117), ligand-induced rotation of the TMDs of rGCs may dissociate the dimeric KHD to adopt relatively flexible structures for ATP binding with positive cooperativity, and this flexibility may allow subunit rotation of the GCD dimer to form a closed, catalytically competent configuration. Such rGC rotational flexibility may explain how GCY-8, GCY-18, and GCY-23 function as thermosensors. The rotational flexibility is likely driven by thermal energy, and temperatures above the growth temperature may induce rotation of the receptor TMD for activation. The temperature thresholds may be determined by phosphorylation states of the receptor KHD. Configuration changes in the KHD dimer may also be induced through interaction with other proteins such as GCAPs and NCS-1 for the activation of rGCs without ligand binding to the receptor ECD, as shown in the ODR-1/DAF-11 heterodimer, GC-E and GC-F. In olfaction and dauer formation in *C. elegans*, GPCRs serve as sensor molecules and regulate cGMP concentrations without involvement of PDEs. Therefore, the Gα subunit of heterotrimeric G proteins may also directly interact with the KHD or the GCD of rGCs to regulate GC activity of the receptor (Figure 1C).

## Conclusion

The *C. elegans* genome encodes 27 rGCs, 6 of which are expressed in a single sensory neuron, ASEL. These rGCs are involved in diverse sensory processing including gustation, thermosensation, olfaction, pheromone sensation, and phototransduction. It has been shown that the homodimeric GCY-14 in ASEL senses alkaline pH, Na^+^, and Li^+^. Therefore, a single sensory neuron serves as a sensor for various environmental cues. Like all mammalian rGCs with a homodimeric structure, most *C. elegans* rGCs act as a homodimer. However, it is now clear that ODR-1 and DAF-11 have an obligate heterodimeric structure. Therefore, other GCYs, such as GCY-4 and GCY-22 in ASER, expressed in the same neuron may also function as heterodimers. It is also apparent that rGCs such as GCY-14, GCY-8, and GCY-9 serve as direct sensor molecules for alkaline pH, temperature, and molecular CO_2_, respectively. As the ECD of ODR-1/DAF-11 and GCY-12 is dispensable, in contrast, these rGCs serve to relay signals from GPCRs, although they may be direct sensors for other environmental cues.

Unlike other type-1 transmembrane receptors, such as receptor tyrosine kinases, rGCs insert the KHD between the TMD and the GCD, which plays a key role in regulation of GC activity through phosphorylation and binding of ATP. Reminiscent of the kinase domain dimer of the epidermal growth factor receptor, this KHD may have a stable homodimeric structure prior to ligand binding to the ECD, in which stability may depend on phosphorylation levels of the domain, to prevent the GCD dimer from taking a flexible, active structure. Ligand binding to the ECD seems to induce rotation (or twist) of the TMD parallel to the plane of the membrane, which may subsequently dissociate the KHD dimer for activation of the GCD. The actual, detailed mechanism of rGC activation by ligand must await determination of the KHD structure, interaction between the linker region and the GCD, and ultimately the structure of full-length rGC with and without bound ligand.

## Author Contributions

The author confirms being the sole contributor of this work and approved it for publication.

## Conflict of Interest Statement

The author declares that the research was conducted in the absence of any commercial or financial relationships that could be construed as a potential conflict of interest.

## References

[1] Ashman DF Lipton R Melicow MM Price TD Isolation of adenosine 3’,5’-monophosphate and guanosine 3’,5’-monophosphate from rat urine. Biochem Biophys Res Commun (1963) 11:330–4. 10.1016/0006-291X(63)90566-7 13965190

[2] Kuhn M. Molecular physiology of membrane guanylyl cyclase receptors. Physiol Rev (2016) 96:751–804. 10.1152/physrev.00022.2015 27030537

[3] Potter LR Guanylyl cyclase structure, function and regulation. Cell Signal (2011) 23:1921–6. 10.1016/j.cellsig.2011.09.001 21914472 PMC4856045

[4] Morton DB. Invertebrates yield a plethora of atypical guanylyl cyclases. Mol Neurobiol (2004) 29:97–116. 10.1385/MN:29:2:097 15126679

[5] Hardman JG Sutherland EW Guanyl cyclase, an enzyme catalyzing the formation of guanosine 3’,5’-monophosphate from guanosine triphosphate. J Biol Chem (1969) 244:6363–70. 4982201

[6] Schultz G Bohme E Munske K Guanyl cyclase. Determination of enzyme activity. Life Sci (1969) 8:1323–32. 10.1016/0024-3205(69)90189-1 5363364

[7] White AA Aurbach GD Detection of guanyl cyclase in mammalian tissues. Biochim Biophys Acta (1969) 191:686–97. 10.1016/0005-2744(69)90362-3 4312210

[8] Kimura H Murad F Evidence for two different forms of guanylate cyclase in rat heart. J Biol Chem (1974) 249:6910–6. 4153733

[9] Garbers DL Gray JP Guanylate cyclase from sperm of the sea urchin, *Strongylocentrotus purpuratus*. Methods Enzymol (1974) 38:196–9. 10.1016/0076-6879(74)38031-7 4156070

[10] Chrisman TD Garbers DL Parks MA Hardman JG. Characterization of particulate and soluble guanylate cyclases from rat lung. J Biol Chem (1975) 250:374–81. 234425

[11] Garbers DL. Sea urchin sperm guanylate cyclase. Purification and loss of cooperativity. J Biol Chem (1976) 251:4071–7. 6469

[12] Gerzer R Bohme E Hofmann F Schultz G Soluble guanylate cyclase purified from bovine lung contains heme and copper. FEBS Lett (1981) 132:71–4. 10.1016/0014-5793(81)80429-2 6117479

[13] Singh S Lowe DG Thorpe DS Rodriguez H Kuang WJ Dangott LJ Membrane guanylate cyclase is a cell-surface receptor with homology to protein kinases. Nature (1988) 334:708–12. 10.1038/334708a0 2901039

[14] Nakane M Saheki S Kuno T Ishii K Murad F. Molecular cloning of a cDNA coding for 70 kilodalton subunit of soluble guanylate cyclase from rat lung. Biochem Biophys Res Commun (1988) 157:1139–47. 10.1016/S0006-291x(88)80992-6 2905128

[15] Koesling D Harteneck C Humbert P Bosserhoff A Frank R Schultz G The primary structure of the larger subunit of soluble guanylyl cyclase from bovine lung: homology between the 2 subunits of the enzyme. FEBS Lett (1990) 266:128–32. 10.1016/0014-5793(90)81523-Q 1973124

[16] Pichlo M Bungert-Plumke S Weyand I Seifert R Bonigk W Strunker T High density and ligand affinity confer ultrasensitive signal detection by a guanylyl cyclase chemoreceptor. J Cell Biol (2014) 207:675. 10.1083/jcb.20140202711112014c 25135936 PMC4137060

[17] Sharma RK Duda T Makino CL. Integrative signaling networks of membrane guanylate cyclases: biochemistry and physiology. Front Mol Neurosci (2016) 9:83. 10.3389/fnmol.2016.00083 27695398 PMC5023690

[18] Kamisaki Y Saheki S Nakane M Palmieri JA Kuno T Chang BY Soluble guanylate cyclase from rat lung exists as a heterodimer. J Biol Chem (1986) 261:7236–41. 2872214

[19] Harteneck C Koesling D Soling A Schultz G Bohme E Expression of soluble guanylyl cyclase: catalytic activity requires 2 enzyme subunits. FEBS Lett (1990) 272:221–3. 10.1016/0014-5793(90)80489-6 1977619

[20] Nighorn A Byrnes KA Morton DB. Identification and characterization of a novel beta subunit of soluble guanylyl cyclase that is active in the absence of a second subunit and is relatively insensitive to nitric oxide. J Biol Chem (1999) 274:2525–31. 10.1074/jbc.274.4.2525 9891024

[21] Koglin M Vehse K Budaeus L Scholz H Behrends S. Nitric oxide activates the beta 2 subunit of soluble guanylyl cyclase in the absence of a second subunit. J Biol Chem (2001) 276:30737–43. 10.1074/jbc.M102549200 11406623

[22] Lucas KA Pitari GM Kazerounian S Ruiz-Stewart I Park J Schulz S Guanylyl cyclases and signaling by cyclic GMP. Pharmacol Rev (2000) 52:375–414. 10977868

[23] Ortiz CO Etchberger JF Posy SL Frokjaer-Jensen C Lockery S Honig B Searching for neuronal left/right asymmetry: genomewide analysis of nematode receptor-type guanylyl cyclases. Genetics (2006) 173:131–49. 10.1534/genetics.106.055749 16547101 PMC1461427

[24] Yu S Avery L Baude E Garbers DL. Guanylyl cyclase expression in specific sensory neurons: a new family of chemosensory receptors. Proc Natl Acad Sci U S A (1997) 94:3384–7. 10.1073/pnas.94.7.3384 9096403 PMC20379

[25] Bargmann CI. Neurobiology of the *Caenorhabditis elegans* genome. Science (1998) 282:2028–33. 10.1126/science.282.5396.2028 9851919

[26] Murayama T Takayama J Fujiwara M Maruyama IN. Environmental alkalinity sensing mediated by the transmembrane guanylyl cyclase GCY-14 in *C. elegans*. Curr Biol (2013) 23:1007–12. 10.1016/j.cub.2013.04.052 23664973

[27] Smith ES Martinez-Velazquez L Ringstad N. A chemoreceptor that detects molecular carbon dioxide. J Biol Chem (2013) 288:37071–81. 10.1074/jbc.M113.517367 24240097 PMC3873563

[28] Takeishi A Yu YV Hapiak VM Bell HW O’Leary T Sengupta P Receptor-type guanylyl cyclases confer thermosensory responses in *C. elegans*. Neuron (2016) 90:235–44. 10.1016/j.neuron.2016.03.002 27041501 PMC4840083

[29] Johnson JL Leroux MR. cAMP and cGMP signaling: sensory systems with prokaryotic roots adopted by eukaryotic cilia. Trends Cell Biol (2010) 20:435–44. 10.1016/j.tcb.2010.05.005 20541938

[30] Liu J Ward A Gao J Dong Y Nishio N Inada H *C. elegans* phototransduction requires a G protein-dependent cGMP pathway and a taste receptor homolog. Nat Neurosci (2010) 13:715–22. 10.1038/nn.2540 20436480 PMC2882063

[31] L’Etoile ND Bargmann CI. Olfaction and odor discrimination are mediated by the *C. elegans* guanylyl cyclase ODR-1. Neuron (2000) 25:575–86. 10.1016/S0896-6273(00)81061-2 10774726

[32] Ludewig AH Schroeder FC Ascaroside signaling in *C. elegans*. WormBook (2013):1–22. 10.1895/wormbook.1.155.1 PMC3758900 23355522

[33] Birnby DA Link EM Vowels JJ Tian H Colacurcio PL Thomas JH. A transmembrane guanylyl cyclase (DAF-11) and Hsp90 (DAF-21) regulate a common set of chemosensory behaviors in *Caenorhabditis elegans*. Genetics (2000) 155:85–104. 10790386 10.1093/genetics/155.1.85 PMC1461074

[34] Ortiz CO Faumont S Takayama J Ahmed HK Goldsmith AD Pocock R Lateralized gustatory behavior of *C. elegans* is controlled by specific receptor-type guanylyl cyclases. Curr Biol (2009) 19:996–1004. 10.1016/j.cub.2009.05.043 19523832 PMC2730525

[35] Smith HK Luo L O’Halloran D Guo D Huang XY Samuel AD Defining specificity determinants of cGMP mediated gustatory sensory transduction in *Caenorhabditis elegans*. Genetics (2013) 194:885–901. 10.1534/genetics.113.152660 23695300 PMC3730918

[36] Johnston RJ Jr Chang S Etchberger JF Ortiz CO Hobert O. microRNAs acting in a double-negative feedback loop to control a neuronal cell fate decision. Proc Natl Acad Sci U S A (2005) 102:12449–54. 10.1073/pnas.0505530102 16099833 PMC1194938

[37] Inada H Ito H Satterlee J Sengupta P Matsumoto K Mori I. Identification of guanylyl cyclases that function in thermosensory neurons of *Caenorhabditis elegans*. Genetics (2006) 172:2239–52. 10.1534/genetics.105.050013 16415369 PMC1456394

[38] Fujiwara M Hino T Miyamoto R Inada H Mori I Koga M The importance of cGMP signaling in sensory cilia for body size regulation in *Caenorhabditis elegans*. Genetics (2015) 201:1497–510. 10.1534/genetics.115.177543 26434723 PMC4676540

[39] Bargmann CI Hartwieg E Horvitz HR. Odorant-selective genes and neurons mediate olfaction in *C. elegans*. Cell (1993) 74:515–27. 10.1016/0092-8674(93)80053-H 8348618

[40] Adachi T Kunitomo H Tomioka M Ohno H Okochi Y Mori I Reversal of salt preference is directed by the insulin/PI3K and Gq/PKC signaling in *Caenorhabditis elegans*. Genetics (2010) 186:1309–19. 10.1534/genetics.110.119768 20837997 PMC2998313

[41] Tsunozaki M Chalasani SH Bargmann CI. A behavioral switch: cGMP and PKC signaling in olfactory neurons reverses odor preference in *C. elegans*. Neuron (2008) 59:959–71. 10.1016/j.neuron.2008.07.038 18817734 PMC2586605

[42] Shinkai Y Yamamoto Y Fujiwara M Tabata T Murayama T Hirotsu T Behavioral choice between conflicting alternatives is regulated by a receptor guanylyl cyclase, GCY-28, and a receptor tyrosine kinase, SCD-2, in AIA interneurons of *Caenorhabditis elegans*. J Neurosci (2011) 31:3007–15. 10.1523/JNEUROSCI.4691-10.2011 21414922 PMC6623760

[43] Komatsu H Mori I Rhee JS Akaike N Ohshima Y. Mutations in a cyclic nucleotide-gated channel lead to abnormal thermosensation and chemosensation in *C. elegans*. Neuron (1996) 17:707–18. 10.1016/j.celrep.2015.11.064 8893027

[44] Coburn CM Bargmann CI. A putative cyclic nucleotide-gated channel is required for sensory development and function in *C. elegans*. Neuron (1996) 17:695–706. 10.1016/S0896-6273(00)80201-9 8893026

[45] Fujiwara M Sengupta P McIntire SL. Regulation of body size and behavioral state of *C. elegans* by sensory perception and the EGL-4 cGMP-dependent protein kinase. Neuron (2002) 36:1091–102. 10.1016/S0896-6273(02)01093-0 12495624

[46] Hirose T Nakano Y Nagamatsu Y Misumi T Ohta H Ohshima Y. Cyclic GMP-dependent protein kinase EGL-4 controls body size and lifespan in *C elegans*. Development (2003) 130:1089–99. 10.1242/dev.00330 12571101

[47] L’Etoile ND Coburn CM Eastham J Kistler A Gallegos G Bargmann CI. The cyclic GMP-dependent protein kinase EGL-4 regulates olfactory adaptation in *C. elegans*. Neuron (2002) 36:1079–89. 10.1016/S0896-6273(02)01066-8 12495623

[48] Kuhara A Inada H Katsura I Mori I. Negative regulation and gain control of sensory neurons by the *C. elegans* calcineurin TAX-6. Neuron (2002) 33:751–63. 10.1016/S0896-6273(02)00607-4 11879652

[49] Gomez M De Castro E Guarin E Sasakura H Kuhara A Mori I Ca^2+^ signaling via the neuronal calcium sensor-1 regulates associative learning and memory in *C. elegans*. Neuron (2001) 30:241–8. 10.1016/S0896-6273(01)00276-8 11343658

[50] Shaye DD Greenwald I. OrthoList: a compendium of *C. elegans* genes with human orthologs. PLoS One (2011) 6:e20085. 10.1371/journal.pone.0020085 21647448 PMC3102077

[51] White JG Southgate E Thomson JN Brenner S. The structure of the nervous system of the nematode *Caenorhabditis elegans*. Philos Trans R Soc Lond B Biol Sci (1986) 314:1–340. 10.1098/rstb.1986.0056 22462104

[52] Hedgecock EM Russell RL. Normal and mutant thermotaxis in the nematode *Caenorhabditis elegans*. Proc Natl Acad Sci U S A (1975) 72:4061–5. 10.1073/pnas.72.10.4061 1060088 PMC433138

[53] Mori I Ohshima Y. Neural regulation of thermotaxis in *Caenorhabditis elegans*. Nature (1995) 376:344–8. 10.1038/376344a0 7630402

[54] Ryu WS Samuel AD. Thermotaxis in *Caenorhabditis elegans* analyzed by measuring responses to defined thermal stimuli. J Neurosci (2002) 22:5727–33. 12097525 10.1523/JNEUROSCI.22-13-05727.2002 PMC6758190

[55] Biron D Shibuya M Gabel C Wasserman SM Clark DA Brown A A diacylglycerol kinase modulates long-term thermotactic behavioral plasticity in *C. elegans*. Nat Neurosci (2006) 9:1499–505. 10.1038/nn1796 17086178

[56] Biron D Wasserman S Thomas JH Samuel AD Sengupta P. An olfactory neuron responds stochastically to temperature and modulates *Caenorhabditis elegans* thermotactic behavior. Proc Natl Acad Sci U S A (2008) 105:11002–7. 10.1073/pnas.0805004105 18667708 PMC2504807

[57] Chi CA Clark DA Lee S Biron D Luo L Gabel CV Temperature and food mediate long-term thermotactic behavioral plasticity by association-independent mechanisms in *C. elegans*. J Exp Biol (2007) 210:4043–52. 10.1242/jeb.006551 17981872

[58] Kuhara A Okumura M Kimata T Tanizawa Y Takano R Kimura KD Temperature sensing by an olfactory neuron in a circuit controlling behavior of *C. elegans*. Science (2008) 320:803–7. 10.1126/science.1148922 18403676

[59] Beverly M Anbil S Sengupta P. Degeneracy and neuromodulation among thermosensory neurons contribute to robust thermosensory behaviors in *Caenorhabditis elegans*. J Neurosci (2011) 31:11718–27. 10.1523/JNEUROSCI.1098-11.2011 21832201 PMC3167209

[60] Ward S Thomson N White JG Brenner S Electron microscopical reconstruction of the anterior sensory anatomy of the nematode *Caenorhabditis elegans*. J Comp Neurol (1975) 160:313–37. 10.1002/cne.901600305 1112927

[61] Ramot D MacInnis BL Goodman MB. Bidirectional temperature-sensing by a single thermosensory neuron in *C. elegans*. Nat Neurosci (2008) 11:908–15. 10.1038/nn.2157 18660808 PMC2587641

[62] Kobayashi K Nakano S Amano M Tsuboi D Nishioka T Ikeda S Single-cell memory regulates a neural circuit for sensory behavior. Cell Rep (2016) 14:11–21. 10.1016/j.celrep.2015.11.064 26725111

[63] Yoshida A Nakano S Suzuki T Ihara K Higashiyama T Mori I. A glial K(+)/Cl(-) cotransporter modifies temperature-evoked dynamics in *Caenorhabditis elegans* sensory neurons. Genes Brain Behav (2016) 15:429–40. 10.1111/gbb.12260 26463820

[64] Nguyen PA Liou W Hall DH Leroux MR. Ciliopathy proteins establish a bipartite signaling compartment in a *C. elegans* thermosensory neuron. J Cell Sci (2014) 127:5317–30. 10.1242/jcs.157610 25335890 PMC4265742

[65] Wasserman SM Beverly M Bell HW Sengupta P. Regulation of response properties and operating range of the AFD thermosensory neurons by cGMP signaling. Curr Biol (2011) 21:353–62. 10.1016/j.cub.2011.01.053 21315599 PMC3057529

[66] Glauser DA Chen WC Agin R Macinnis BL Hellman AB Garrity PA Heat avoidance is regulated by transient receptor potential (TRP) channels and a neuropeptide signaling pathway in *Caenorhabditis elegans*. Genetics (2011) 188:91–103. 10.1534/genetics.111.127100 21368276 PMC3120139

[67] Wang D O’Halloran D Goodman MB. GCY-8, PDE-2, and NCS-1 are critical elements of the cGMP-dependent thermotransduction cascade in the AFD neurons responsible for *C. elegans* thermotaxis. J Gen Physiol (2013) 142:437–49. 10.1085/jgp.201310959 24081984 PMC3787776

[68] Singhvi A Liu B Friedman CJ Fong J Lu Y Huang XY A Glial K/Cl transporter controls neuronal receptive ending shape by chloride inhibition of an rGC. Cell (2016) 165:936–48. 10.1016/j.cell.2016.03.026 27062922 PMC4860081

[69] Scott K. Out of thin air: sensory detection of oxygen and carbon dioxide. Neuron (2011) 69:194–202. 10.1016/j.neuron.2010.12.018 21262460 PMC3038919

[70] Ma DK Ringstad N. The neurobiology of sensing respiratory gases for the control of animal behavior. Front Biol (Beijing) (2012) 7:246–53. 10.1007/s11515-012-1219-x 22876258 PMC3412401

[71] Hallem EA Sternberg PW. Acute carbon dioxide avoidance in *Caenorhabditis elegans*. Proc Natl Acad Sci U S A (2008) 105:8038–43. 10.1073/pnas.0707469105 18524955 PMC2430355

[72] Hallem EA Spencer WC McWhirter RD Zeller G Henz SR Ratsch G Receptor-type guanylate cyclase is required for carbon dioxide sensation by *Caenorhabditis elegans*. Proc Natl Acad Sci U S A (2011) 108:254–9. 10.1073/pnas.1017354108 21173231 PMC3017194

[73] Troemel ER Sagasti A Bargmann CI. Lateral signaling mediated by axon contact and calcium entry regulates asymmetric odorant receptor expression in *C. elegans*. Cell (1999) 99:387–98. 10.1016/S0092-8674(00)81525-1 10571181

[74] Wes PD Bargmann CI. *C. elegans* odour discrimination requires asymmetric diversity in olfactory neurons. Nature (2001) 410:698–701. 10.1038/35070581 11287957

[75] Sengupta P Chou JH Bargmann CI. *odr-10* encodes a seven transmembrane domain olfactory receptor required for responses to the odorant diacetyl. Cell (1996) 84:899–909. 10.1016/S0092-8674(00)81068-5 8601313

[76] Troemel ER Chou JH Dwyer ND Colbert HA Bargmann CI. Divergent seven transmembrane receptors are candidate chemosensory receptors in *C. elegans*. Cell (1995) 83:207–18. 10.1016/0092-8674(95)90162-0 7585938

[77] Lans H Rademakers S Jansen G. A network of stimulatory and inhibitory Galpha-subunits regulates olfaction in *Caenorhabditis elegans*. Genetics (2004) 167:1677–87. 10.1534/genetics.103.024786 15342507 PMC1470997

[78] Roayaie K Crump JG Sagasti A Bargmann CI. The G alpha protein ODR-3 mediates olfactory and nociceptive function and controls cilium morphogenesis in *C. elegans* olfactory neurons. Neuron (1998) 20:55–67. 10.1016/S0896-6273(00)80434-1 9459442

[79] Jansen G Thijssen KL Werner P van der Horst M Hazendonk E Plasterk RH. The complete family of genes encoding G proteins of *Caenorhabditis elegans*. Nat Genet (1999) 21:414–9. 10.1038/7753 10192394

[80] Chalasani SH Chronis N Tsunozaki M Gray JM Ramot D Goodman MB Dissecting a circuit for olfactory behaviour in *Caenorhabditis elegans*. Nature (2007) 450:63–70. 10.1038/nature06292 17972877

[81] Yang XL. Characterization of receptors for glutamate and GABA in retinal neurons. Prog Neurobiol (2004) 73:127–50. 10.1016/j.pneurobio.2004.04.002 15201037

[82] O’Halloran DM Hamilton OS Lee JI Gallegos M L’Etoile ND. Changes in cGMP levels affect the localization of EGL-4 in AWC in *Caenorhabditis elegans*. PLoS One (2012) 7:e31614. 10.1371/journal.pone.0031614 22319638 PMC3272044

[83] O’Halloran DM Altshuler-Keylin S Lee JI L’Etoile ND. Regulators of AWC-mediated olfactory plasticity in *Caenorhabditis elegans*. PLoS Genet (2009) 5:e1000761. 10.1371/journal.pgen.1000761 20011101 PMC2780698

[84] Lee JI O’Halloran DM Eastham-Anderson J Juang BT Kaye JA Scott Hamilton O Nuclear entry of a cGMP-dependent kinase converts transient into long-lasting olfactory adaptation. Proc Natl Acad Sci U S A (2010) 107:6016–21. 10.1073/pnas.1000866107 20220099 PMC2851914

[85] Juang BT Gu C Starnes L Palladino F Goga A Kennedy S Endogenous nuclear RNAi mediates behavioral adaptation to odor. Cell (2013) 154:1010–22. 10.1016/j.cell.2013.08.006 23993094 PMC4274153

[86] Cho SW Cho JH Song HO Park CS. Identification and characterization of a putative cyclic nucleotide-gated channel, CNG-1, in *C. elegans*. Mol Cells (2005) 19:149–54. 15750353

[87] Cassada RC Russell RL The dauerlarva, a post-embryonic developmental variant of the nematode *Caenorhabditis elegans*. Dev Biol (1975) 46:326–42. 10.1016/0012-1606(75)90109-8 1183723

[88] Kimura KD Tissenbaum HA Liu Y Ruvkun G daf-2, an insulin receptor-like gene that regulates longevity and diapause in *Caenorhabditis elegans*. Science (1997) 277:942–6. 10.1126/science.277.5328.942 9252323

[89] Ren P Lim CS Johnsen R Albert PS Pilgrim D Riddle DL. Control of *C. elegans* larval development by neuronal expression of a TGF-beta homolog. Science (1996) 274:1389–91. 10.1126/science.274.5291.1389 8910282

[90] Schackwitz WS Inoue T Thomas JH. Chemosensory neurons function in parallel to mediate a pheromone response in *C. elegans*. Neuron (1996) 17:719–28. 10.1016/S0896-6273(00)80203-2 8893028

[91] Henderson ST Johnson TE. *daf-16* integrates developmental and environmental inputs to mediate aging in the nematode *Caenorhabditis elegans*. Curr Biol (2001) 11:1975–80. 10.1016/S0960-9822(01)00594-2 11747825

[92] Lee R Kermani P Teng KK Hempstead BL. Regulation of cell survival by secreted proneurotrophins. Science (2001) 294:1945–8. 10.1126/science.1065057 11729324

[93] Lin K Hsin H Libina N Kenyon C Regulation of the *Caenorhabditis elegans* longevity protein DAF-16 by insulin/IGF-1 and germline signaling. Nat Genet (2001) 28:139–45. 10.1038/88850 11381260

[94] Kenyon C Chang J Gensch E Rudner A Tabtiang R. A *C. elegans* mutant that lives twice as long as wild type. Nature (1993) 366:461–4. 10.1038/366461a0 8247153

[95] Larsen PL Albert PS Riddle DL. Genes that regulate both development and longevity in *Caenorhabditis elegans*. Genetics (1995) 139:1567–83. 7789761 10.1093/genetics/139.4.1567 PMC1206485

[96] Georgi LL Albert PS Riddle DL *daf-1*, a *C. elegans* gene controlling dauer larva development, encodes a novel receptor protein kinase. Cell (1990) 61:635–45. 10.1016/0092-8674(90)90475-T 2160853

[97] Gunther CV Georgi LL Riddle DLA *Caenorhabditis elegans* type I TGF beta receptor can function in the absence of type II kinase to promote larval development. Development (2000) 127:3337–47. 10887089 10.1242/dev.127.15.3337

[98] Estevez M Attisano L Wrana JL Albert PS Massague J Riddle DL. The *daf-4* gene encodes a bone morphogenetic protein receptor controlling *C. elegans* dauer larva development. Nature (1993) 365:644–9. 10.1038/365644a0 8413626

[99] Bargmann CI Horvitz HR. Control of larval development by chemosensory neurons in *Caenorhabditis elegans*. Science (1991) 251:1243–6. 10.1126/science.2006412 2006412

[100] Ailion M Thomas JH. Dauer formation induced by high temperatures in *Caenorhabditis elegans*. Genetics (2000) 156:1047–67. 11063684 10.1093/genetics/156.3.1047 PMC1461313

[101] Thomas JH Birnby DA Vowels JJ. Evidence for parallel processing of sensory information controlling dauer formation in *Caenorhabditis elegans*. Genetics (1993) 134:1105–17. 8375650 10.1093/genetics/134.4.1105 PMC1205579

[102] Gottlieb S Ruvkun G. daf-2, daf-16 and daf-23: genetically interacting genes controlling Dauer formation in *Caenorhabditis elegans*. Genetics (1994) 137:107–20. 8056303 10.1093/genetics/137.1.107 PMC1205929

[103] Murakami M Koga M Ohshima Y. DAF-7/TGF-beta expression required for the normal larval development in *C. elegans* is controlled by a presumed guanylyl cyclase DAF-11. Mech Dev (2001) 109:27–35. 10.1016/S0925-4773(01)00507-X 11677050

[104] McGrath PT Xu Y Ailion M Garrison JL Butcher RA Bargmann CI. Parallel evolution of domesticated *Caenorhabditis* species targets pheromone receptor genes. Nature (2011) 477:321–5. 10.1038/nature10378 21849976 PMC3257054

[105] Park D O’Doherty I Somvanshi RK Bethke A Schroeder FC Kumar U Interaction of structure-specific and promiscuous G-protein-coupled receptors mediates small-molecule signaling in *Caenorhabditis elegans*. Proc Natl Acad Sci U S A (2012) 109:9917–22. 10.1073/pnas.1202216109 22665789 PMC3382479

[106] Daniels SA Ailion M Thomas JH Sengupta P. *egl-4* acts through a transforming growth factor-beta/SMAD pathway in *Caenorhabditis elegans* to regulate multiple neuronal circuits in response to sensory cues. Genetics (2000) 156:123–41. 10978280 10.1093/genetics/156.1.123 PMC1461244

[107] Raizen DM Cullison KM Pack AI Sundaram MV. A novel gain-of-function mutant of the cyclic GMP-dependent protein kinase *egl-4* affects multiple physiological processes in *Caenorhabditis elegans*. Genetics (2006) 173:177–87. 10.1534/genetics.106.057380 16547093 PMC1461420

[108] Edwards SL Charlie NK Milfort MC Brown BS Gravlin CN Knecht JE A novel molecular solution for ultraviolet light detection in *Caenorhabditis elegans*. PLoS Biol (2008) 6:e198. 10.1371/journal.pbio.0060198 18687026 PMC2494560

[109] Ward A Liu J Feng Z Xu XZ. Light-sensitive neurons and channels mediate phototaxis in *C. elegans*. Nat Neurosci (2008) 11:916–22. 10.1038/nn.2155 18604203 PMC2652401

[110] Gong J Yuan Y Ward A Kang L Zhang B Wu Z The *C. elegans* taste receptor homolog LITE-1 is a photoreceptor. Cell (2016) 167:1252–63.e10. 10.1016/j.cell.2016.10.053 27863243 PMC5388352

[111] Lewis JA Hodgkin JA. Specific neuroanatomical changes in chemosensory mutants of the nematode *Caenorhabditis elegans*. J Comp Neurol (1977) 172:489–510. 10.1002/cne.901720306 838889

[112] Fujiwara M Ishihara T Katsura I. A novel WD40 protein, CHE-2, acts cell-autonomously in the formation of *C. elegans* sensory cilia. Development (1999) 126:4839–48. 10518500 10.1242/dev.126.21.4839

[113] Lozano E Saez AG Flemming AJ Cunha A Leroi AM. Regulation of growth by ploidy in *Caenorhabditis elegans*. Curr Biol (2006) 16:493–8. 10.1016/j.cub.2006.01.048 16527744

[114] Tain LS Lozano E Saez AG Leroi AM. Dietary regulation of hypodermal polyploidization in *C. elegans*. BMC Dev Biol (2008) 8:28. 10.1186/1471-213X-8-28 18366811 PMC2275723

[115] Fujiwara M Teramoto T Ishihara T Ohshima Y McIntire SL. A novel zf-MYND protein, CHB-3, mediates guanylyl cyclase localization to sensory cilia and controls body size of *Caenorhabditis elegans*. PLoS Genet (2010) 6:e1001211. 10.1371/journal.pgen.1001211 21124861 PMC2991246

[116] Maruyama IN. Mechanisms of activation of receptor tyrosine kinases: monomers or dimers. Cells (2014) 3:304–30. 10.3390/cells3020304 24758840 PMC4092861

[117] Maruyama IN Activation of transmembrane cell-surface receptors via a common mechanism? The “rotation model”. Bioessays (2015) 37:959–67. 10.1002/bies.201500041 26241732 PMC5054922

[118] Chinkers M Wilson EM. Ligand-independent oligomerization of natriuretic peptide receptors. Identification of heteromeric receptors and a dominant negative mutant. J Biol Chem (1992) 267:18589–97. 1382057

[119] van den Akker F Zhang X Miyagi M Huo X Misono KS Yee VC. Structure of the dimerized hormone-binding domain of a guanylyl-cyclase-coupled receptor. Nature (2000) 406:101–4. 10.1038/35017602 10894551

[120] Ogawa H Qiu Y Ogata CM Misono KS. Crystal structure of hormone-bound atrial natriuretic peptide receptor extracellular domain: rotation mechanism for transmembrane signal transduction. J Biol Chem (2004) 279:28625–31. 10.1074/jbc.M313222200 15117952

[121] Misono KS. Atrial natriuretic factor binding to its receptor is dependent on chloride concentration: a possible feedback-control mechanism in renal salt regulation. Circ Res (2000) 86:1135–9. 10.1161/01.RES.86.11.1135 10850964

[122] Kurose H Inagami T Ui M. Participation of adenosine 5’-triphosphate in the activation of membrane-bound guanylate cyclase by the atrial natriuretic factor. FEBS Lett (1987) 219:375–9. 10.1016/0014-5793(87)80256-9 2886366

[123] Chinkers M Singh S Garbers DL. Adenine nucleotides are required for activation of rat atrial natriuretic peptide receptor/guanylyl cyclase expressed in a baculovirus system. J Biol Chem (1991) 266:4088–93. 1671858

[124] Joubert S Jossart C McNicoll N De Lean A. Atrial natriuretic peptide-dependent photolabeling of a regulatory ATP-binding site on the natriuretic peptide receptor-A. FEBS J (2005) 272:5572–83. 10.1111/j.1742-4658.2005.04952.x 16262696

[125] Potter LR Hunter T. Phosphorylation of the kinase homology domain is essential for activation of the A-type natriuretic peptide receptor. Mol Cell Biol (1998) 18:2164–72. 10.1128/MCB.18.4.2164 9528788 PMC121455

[126] Schroter J Zahedi RP Hartmann M Gassner B Gazinski A Waschke J Homologous desensitization of guanylyl cyclase A, the receptor for atrial natriuretic peptide, is associated with a complex phosphorylation pattern. FEBS J (2010) 277:2440–53. 10.1111/j.1742-4658.2010.07658.x 20456499 PMC2901513

[127] Potter LR Garbers DL. Dephosphorylation of the guanylyl cyclase-A receptor causes desensitization. J Biol Chem (1992) 267:14531–4. 1353076

[128] Joubert S Labrecque J De Lean A. Reduced activity of the NPR-A kinase triggers dephosphorylation and homologous desensitization of the receptor. Biochemistry (2001) 40:11096–105. 10.1021/bi010580s 11551207

[129] Larose L McNicoll N Ong H De Lean A. Allosteric modulation by ATP of the bovine adrenal natriuretic factor R1 receptor functions. Biochemistry (1991) 30:8990–5. 10.1021/bi00101a012 1654083

[130] Jewett JR Koller KJ Goeddel DV Lowe DG Hormonal induction of low affinity receptor guanylyl cyclase. EMBO J (1993) 12:769–77. 8095019 10.1002/j.1460-2075.1993.tb05711.x PMC413264

[131] Duda T Venkataraman V Ravichandran S Sharma RK. ATP-regulated module (ARM) of the atrial natriuretic factor receptor guanylate cyclase. Peptides (2005) 26:969–84. 10.1016/j.peptides.2004.08.032 15911066

[132] Chinkers M Garbers DL The protein kinase domain of the ANP receptor is required for signaling. Science (1989) 245:1392–4. 10.1126/science.2571188 2571188

[133] Saha S Biswas KH Kondapalli C Isloor N Visweswariah SS. The linker region in receptor guanylyl cyclases is a key regulatory module: mutational analysis of guanylyl cyclase C. J Biol Chem (2009) 284:27135–45. 10.1074/jbc.M109.020032 19648115 PMC2786029

[134] Winger JA Derbyshire ER Lamers MH Marletta MA Kuriyan J The crystal structure of the catalytic domain of a eukaryotic guanylate cyclase. BMC Struct Biol (2008) 8:42 10.1186/1472-6807-8-42 18842118 PMC2576301

[135] Rauch A Leipelt M Russwurm M Steegborn C. Crystal structure of the guanylyl cyclase Cya2. Proc Natl Acad Sci U S A (2008) 105:15720–5. 10.1073/pnas.0808473105 18840690 PMC2572937

[136] Allerston CK von Delft F Gileadi O. Crystal structures of the catalytic domain of human soluble guanylate cyclase. PLoS One (2013) 8:e57644. 10.1371/journal.pone.0057644 23505436 PMC3591389

[137] Linder JU Schultz JE. The class III adenylyl cyclases: multi-purpose signalling modules. Cell Signal (2003) 15:1081–9. 10.1016/S0898-6568(03)00130-X 14575863

[138] Zhang Y Chou JH Bradley J Bargmann CI Zinn K. The *Caenorhabditis elegans* seven-transmembrane protein ODR-10 functions as an odorant receptor in mammalian cells. Proc Natl Acad Sci U S A (1997) 94:12162–7. 10.1073/pnas.94.22.12162 9342380 PMC23737

[139] Tesmer JJ Sunahara RK Gilman AG Sprang SR. Crystal structure of the catalytic domains of adenylyl cyclase in a complex with Gsalpha.GTPgammaS. Science (1997) 278:1907–16. 10.1126/science.278.5345.1907 9417641

[140] Misono KS Philo JS Arakawa T Ogata CM Qiu Y Ogawa H Structure, signaling mechanism and regulation of the natriuretic peptide receptor guanylate cyclase. FEBS J (2011) 278:1818–29. 10.1111/j.1742-4658.2011.08083.x 21375693 PMC3097287

[141] Parat M Blanchet J De Lean A. Role of juxtamembrane and transmembrane domains in the mechanism of natriuretic peptide receptor A activation. Biochemistry (2010) 49:4601–10. 10.1021/bi901711w 20214400

[142] Jura N Endres NF Engel K Deindl S Das R Lamers MH Mechanism for activation of the EGF receptor catalytic domain by the juxtamembrane segment. Cell (2009) 137:1293–307. 10.1016/j.cell.2009.04.025 19563760 PMC2814540

[143] Stamos J Sliwkowski MX Eigenbrot C. Structure of the epidermal growth factor receptor kinase domain alone and in complex with a 4-anilinoquinazoline inhibitor. J Biol Chem (2002) 277:46265–72. 10.1074/jbc.M207135200 12196540

